# Ecophysiological responses of *Nothofagus obliqua* forests to recent climate drying across the Mediterranean-Temperate biome transition in south-central Chile

**DOI:** 10.1029/2022JG007293

**Published:** 2022-12-18

**Authors:** Rocío Urrutia-Jalabert, Jonathan Barichivich, Paul Szejner, Vicente Rozas, Antonio Lara

**Affiliations:** a Departamento de Ciencias Naturales y Tecnología, Universidad de Aysén, Coyhaique, Chile; b Laboratorio de Dendrocronología y Cambio Global, Instituto de Conservación, Biodiversidad y Territorio, Facultad de Ciencias Forestales y Recursos Naturales, Universidad Austral de Chile, Valdivia, Chile; c Centro de Ciencia del Clima y la Resiliencia, CR2, Santiago, Chile; d Laboratoire des Sciences du Climat et de l’Environnement, IPSL, CRNS/CEA/UVSQ, France; e Instituto de Geografía, Pontificia Universidad Católica de Valparaíso, Valparaíso, Chile; f Departamento de Ciencias Ambientales y del suelo, Instituto de Geología, Universidad Nacional Autónoma de México. Ciudad Universitaria CDMX, México; g iuFOR-EiFAB, Á*rea de Bot*ánica, Campus Duques de Soria, Universidad de Valladolid, 42004 Soria, Spain; h Fundación Centro de los Bosques Nativos FORECOS, Valdivia, Chile

## Abstract

The forests of south-central Chile are facing a drying climate and a megadrought that started in 2010. This study addressed the physiological responses of five *Nothofagus obliqua* stands across the Mediterranean-Temperate gradient (35.9 ° -40.3° S) using carbon isotope discrimination (Δ^13^ C) and intrinsic water use efficiency (iWUE) in tree rings during 1967-2017. Moreover, δ^18^O was evaluated in the northernmost site to better understand the effects of the megadrought in this drier location. These forests have become more efficient in their use of water. However, trees from the densest stand are discriminating more against ^13^C, probably due to reduced photosynthetic rates associated with increasing competition. The strongest associations between climate and Δ^13^C were found in the northernmost stand, suggesting that warmer and drier conditions could have reduced ^13^C discrimination. Tree growth in this site has not decreased, and δ^18^O was negatively related to annual rainfall. However, a shift in this relationship was found since 2007, when both precipitation and δ^18^O decreased, while correlations between δ^18^O and growth increased. This implies that tree growth and δ^18^O are coupled in recent years, but precipitation is not the cause, suggesting that trees probably changed their water source to deeper and more depleted pools. Our research demonstrates that forests are not reducing their growth in central Chile, mainly due to a shift towards the use of deeper water sources. Despite a common climate trend across the gradient, there is a non-uniform response of *N. obliqua* forests to climate drying, being their response site specific. Keywords: Tree rings, stable isotopes, tree physiology, climate gradient, megadrought, climate change.

## 1 Introduction

Rising CO_2_ concentrations have stimulated productivity (Granda et al., 2017; Huang et al., 2017; Martínez-Vilalta et al., 2008; Soule & Knapp, 2006) and water use efficiency in many forested ecosystems worldwide (Andreu-Hayles et al., 2011; Arco Molina et al., 2019; Gómez-Guerrero et al., 2013; Van Der Sleen et al., 2015; Tognetti et al., 2014; Urrutia-Jalabert et al., 2015). However, the increased risk of low precipitation and heat waves, and the more frequent occurrence of intense and severe droughts associated to global warming (Chiang et al., 2021; Morales et al., 2020), have led to decreased growth, die-off events and a high vulnerability of forests to climatically-induced tree mortality (Adams et al., 2017; Allen et al., 2010; Allen et al., 2015; Anderegg et al., 2019; Szejner et al., 2020; Zhao & Running, 2010).

Several studies have reported how changes in climate conditions and drought occurrence have reduced tree growth and forest productivity in temperate regions of the Northern Hemisphere (e.g: Cailleret et al., 2017; Cavin and Jump, 2017; Gazol et al., 2017; Gazol et al., 2017; Gazol et al., 2018; Serra-Maluquer et al., 2019), and in southern South America (e.g: Christie et al., 2011; Fajardo et al., 2019; Rodríguez-Catón et al., 2015; Suarez et al., 2015; Urrutia-Jalabert et al., 2015; Venegas-González et al., 2019, 2018; Villalba et al., 2012). Typically, radial growth of widely distributed tree species tends to be more sensitive to drought in equatorward than poleward populations, but sometimes the opposite can occur (Camarero et al., 2021). Mediterranean forests have shown a higher capacity to recover their growth after a drought than temperate forests, which have a lower recovery, but tend to resist droughts better (Antonio Gazol et al., 2018; Urrutia-Jalabert et al., 2021).

The impacts of changing environmental conditions on tree growth and physiological responses of forests over time can be assessed by quantifying annual tree-ring growth and stable isotopes integrated in tree-ring cellulose. Measurements of the stable isotopes of carbon (δ^13^C) and oxygen (δ^18^O) in wood cellulose are useful to detect and understand the physiological drivers of changes in intrinsic water use efficiency in forests through time (Guerrieri et al., 2019; Lévesque et al., 2014). Intrinsic Water use Efficiency (iWUE) is the relationship between carbon assimilation (A) and stomatal conductance (gs) (Farquhar et al., 1989; Farquhar et al., 1982). Several studies have reported positive trends in iWUE in forests around the world over the past few decades (Camarero and Fajardo, 2017; Franks et al., 2013; Guerrieri et al., 2019; Lavergne et al., 2016; Lévesque et al., 2014; Urrutia-Jalabert et al., 2015). These positive trends in iWUE can be attributed to an increase in photosynthetic rates due to rising atmospheric CO_2_, a decrease in stomatal conductance, or a combination of both mechanisms (Andreu-Hayles et al., 2011). It has been shown that in mesic forests the increase of photosynthetic rates with rising CO_2_ has mostly driven the increase in water use efficiency, with stomatal conductance reductions limited to those species that face moisture limitations (Guerrieri et al., 2019). In the case of the temperate forests of southern South America, different studies have found mostly an increase in iWUE over the last few decades, which was attributed to either physiological mechanism (Arco Molina et al., 2019; Fajardo et al., 2019; Lavergne et al., 2016; Puchi et al., 2021; Urrutia-Jalabert et al., 2015).

Changes in iWUE can be better disentangled when δ^18^O is jointly analyzed, since it varies independently of carbon isotope fractionation during photosynthesis (Roden et al., 2022) and can be a proxy for gs under certain environmental conditions if the effect of source water signals is accounted for. In this setting, an increase in both δ^13^C and δ^18^O through time suggests that increases in iWUE are driven by decreased stomatal conductance rather by increased photosynthetic activity rates, since δ^18^O can be negatively related to gs (Farquhar & Lloyd, 1993; Roden et al., 2022). Although the environmental and physiological drivers of δ^18^O variability of forest trees might be complex to disentangle empirically, δ^18^O also carries information about the water sources used by trees through time (Sargeant et al., 2019). Thus, combining both tree-ring isotopes with radial growth can help disentangling leaf-level and belowground physiological adjustments experienced by forests under climate change (Barichivich et al., 2021; Castruita-Esparza et al., 2019).

The mid-latitudes of the Southern Hemisphere are experiencing a gradual process of subtropical dryland expansion with climate change (Villamayor et al., 2021). The temperate and Mediterranean Andes of South America are located right on this sensitive region of change, which marks the biome transition from Mediterranean to Temperate climate in Chile. The region has experienced a moderate warming and a sustained precipitation decline during recent decades, linked to changes in tropical and high-latitude atmospheric circulation (Falvey and Garreaud, 2009; Gonzalez-Reyes and Muñoz, 2013; Lara et al., 2020; Morales et al., 2020; Trenberth et al., 2007; Urrutia-Jalabert et al., 2015; Villamayor et al., 2021). The most severe and long-lasting drought over the last millennium (locally known as megadrought) has been ongoing since 2010 (Barichivich et al., 2020; Garreaud et al., 2020), with average precipitation deficits ranging between 25-45% and up to 80% in some places during 2019 (SEGRA, 2020). This unprecedented megadrought has had catastrophic effects on forest ecosystems (Miranda et al., 2020; Venegas-González et al., 2022), fire regimes (Bowman et al., 2019), riverflow and water reservoir levels (Barría et al., 2021).

A recent study across the Mediterranean-Temperate climate transition in south-central Chile found that radial growth of a widely distributed deciduous tree species of southern beech (*Nothofagus obliqua*), was positively related to precipitation and negatively related to maximum temperature along most of the gradient (Urrutia-Jalabert et al., 2021). Moreover, the two northernmost, more Mediterranean stands were among the most resilient to repeated droughts and tree growth in these sites had not been strongly affected by the current megadrought (Urrutia-Jalabert et al., 2021). This suggests that Mediterranean populations of the species may have different functional responses to adapt to drying and warming conditions than temperate populations.

Research focused on assessing the functional response of forests to climate change using carbon and oxygen stable isotopes of wood cellulose in Chile, is very scarce. Therefore, conducting this type of studies brings an opportunity to better understand the effects of climate change on these transitional forests. The present study aims to go beyond what tree growth alone can reveal, evaluating physiological and functional changes in five of the nine *Nothofagus obliqua* second-growth stands studied by Urrutia-Jalabert et al., (2021). These changes were assessed by quantifying carbon isotope discrimination in annual tree rings and their associated iWUE through time. Additionally, oxygen isotopes were also analyzed for the northernmost and drier site to understand the functional behavior of the species under Mediterranean conditions. The specific objectives of this study were to: i) evaluate changes in carbon isotope discrimination and iWUE in five *Nothofagus obliqua* secondary stands across the Mediterranean-Temperate gradient in south-central Chile during recent decades, including the megadrought period; ii) assess the physiological behavior of a Mediterranean stand by evaluating the oxygen isotope composition recorded in tree rings during recent decades, including the megadrought period and iii) evaluate which are the main climate variables related to the physiological performance of *Nothofagus* stands across the Mediterranean-Temperate region.

## 2 Methods

### 2.1 Study sites

**Figure 1:**
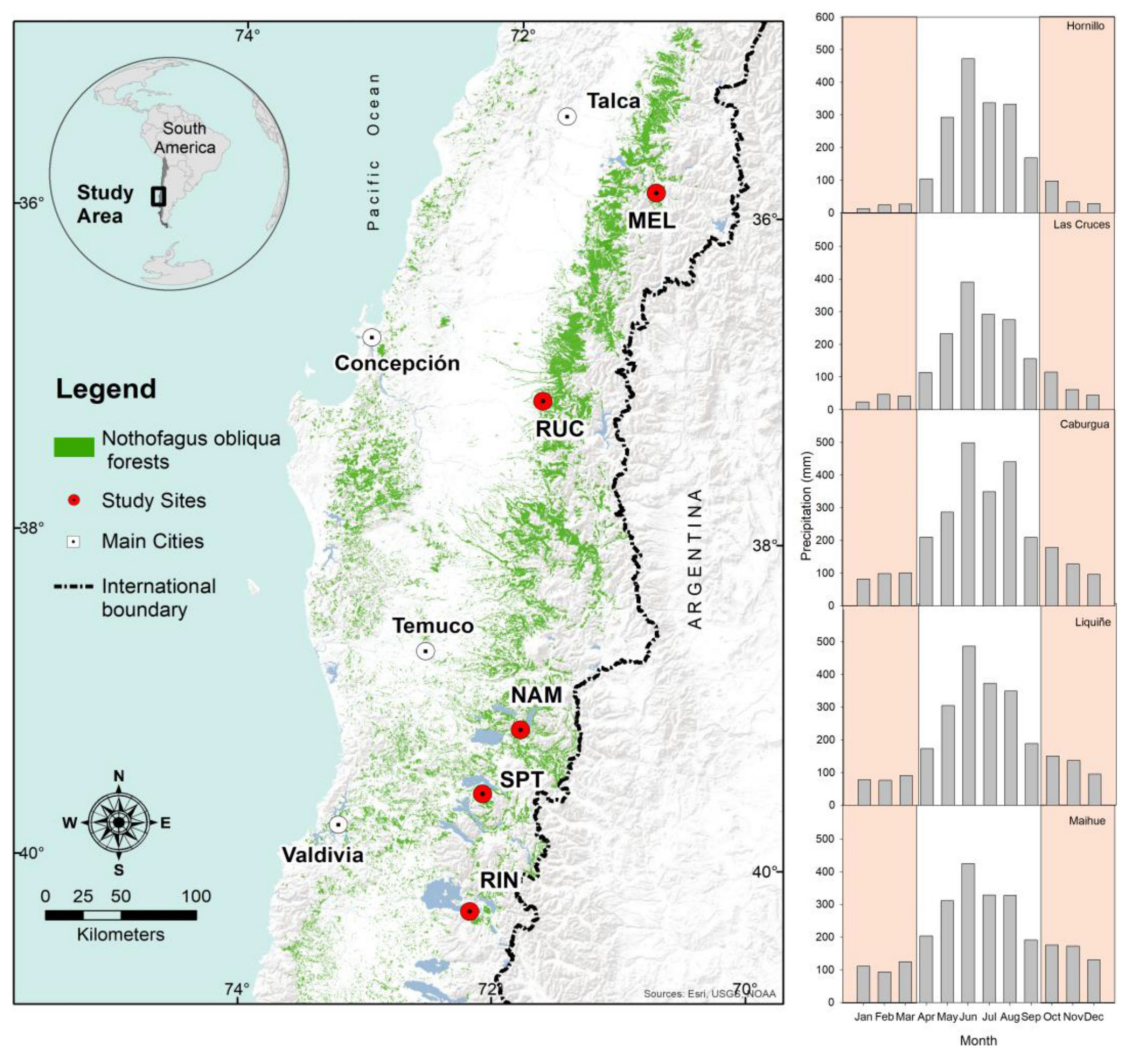
Left: Map showing the distribution of *Nothofagus obliqua* forests and the location of the five study sites: MEL (Melado), RUC (Rucamanqui), NAM (Namuncai), SPT (San Pablo de Tregua), RIN (Riñinahue). Right: monthly precipitation distribution at the closest meteorological station to each study site (period 1995-2015), from north to south. The six months with lower precipitation (October-March) are shown with orange shading.

Five secondary and even-aged stands along a latitudinal gradient of almost five degrees in south-central Chile (35.9° to 40.3° S) were assessed in this study. These stands were selected among the nine stands used previously by Urrutia-Jalabert et al., (2021) to study the resilience of drought to droughts. The selected stands were from north to south: Melado (MEL, 35.9° S) in Maule Region, Rucamanqui (RUC, 36.2° S) in Bío Bío Region, Namuncai (NAM, 39.2° S) in Araucanía Region, and San Pablo de Tregua (SPT, 39.6° S) and Riñinahue (RIN, 40.3° S) in Los Ríos Region (Figure 1 and Table S1 for site characteristics). The stands covered an elevation range from 156 (RIN) to 1168 m a.s.l (MEL) and were mostly dominated by *N. obliqua,* accounting for more than 70% of the total basal area (Table S1, Donoso et al., 1993; Gezan et al., 2007). Stand densities varied between 390 trees/ha in MEL and 1100 trees/ha in RUC (Table S1, Urrutia-Jalabert et al., 2021). Climate in the area ranged from Mediterranean in the north to Temperate with some Mediterranean summer influence in the south, with differences in rainfall mostly given by the amount of precipitation falling during spring-summer (20% and 38% in the north and south, respectively) and summer (4.7% and 17% in the north and south, respectively, Table S1, Urrutia-Jalabert et al., 2021).

### 2.2 Tree-ring stable isotopes chronologies

Five cores per site (MEL, RUC, NAM, SPT and RIN) were selected for the isotopic analyses from the tree-ring chronologies developed by Urrutia-Jalabert et al., (2021). These tree-ring chronologies were developed by standardizing the raw tree-ring width measurements with a negative exponential curve or a regression line using the ARSTAN44xp program (Cook & Krusic, 2005; Urrutia-Jalabert et al., 2021). The maximum length of tree-ring chronologies, which were a good representation of stand age (cores up to the bole center), were the following: 177, 62, 72, 62 and 51 years for MEL, RUC, NAM, SPT and RIN, respectively (Table S1, Urrutia-Jalabert et al., 2021). Annual rings for the common period 1967-2017 were cut using a razor blade under a stereo microscope. At each site, rings from the same year were pooled for cellulose extraction. Carbon and oxygen isotopic analysis of the cellulose was carried out on a Thermo Delta V isotope ratio mass spectrometer (IRMS) interfaced to a NC2500 elemental analyzer for Carbon and to a Temperature Conversion Elemental Analyzer (TC/EA) for Oxygen, at the Cornell University Stable Isotope Laboratory (COIL).

The carbon isotope ratio (^13^C/^12^C) or δ^13^C was expressed in per mill with reference to a standard with a known isotopic ratio. The δ^13^C was expressed as:

$$\delta^{13}\text{C}(∴)=\frac{\left(R\text{sample}-1\right)}{R\text{standard}}\times 1000$$

R_sample_ is the carbon ratio of every sample and Rstandard is the carbon ratio corresponding to the standard Vienna Pee Dee Belemnite or VPDB (Mccarroll & Loader, 2004). The precision of the instrument, measured with an internal plant standard was *0.007‰.* The carbon isotopic discrimination (Δ‰) that results from the preferential election of ^12^C over ^13^C during photosynthesis was obtained through the formula of Farquhar et al., (1982):

$$\Delta^{13}C=\frac{\delta^{13}C_{\text{atm}}-\delta^{13}C_{\text{tree}}}{1+\frac{\delta^{13}C_{\text{tree}}}{1000}}$$

δ^13^Catm and δ^13^Ctree correspond to the isotopic value of atmospheric CO_2_ and the ratio measured in tree rings, respectively. Carbon discrimination is linearly related to the ratio of intercellular (*ci*) to ambient (*ca*) CO_2_ concentration as follows (Farquhar et al., 1982):

$$Ci=\frac{\Delta^{13}\text{C}-\text{a}}{\text{b}-\text{a}}*ca$$

Where Δ^13^C is the isotopic discrimination of the sample estimated with the equation above, the parameter “a” is the fractionation associated with CO_2_ diffusing through stomata (4.4 ‰), “b” is the fractionation associated with the RuBisCO enzyme (27 ‰) and *ca* is the annual atmospheric CO_2_ concentration. Finally, using the estimated ci, the intrinsic water use efficiency (iWUE) can be calculated as the ratio of assimilation to stomatal conductance as follows:

$$i\text{WUE}=\frac{\text{A}}{g_{s}}=\frac{g_{CO_{2}}(ca-ci)}{g_{s}}=\frac{ca-ci}{1.6}$$

Where A is carbon assimilation, g_s_ is stomatal conductance of water, g_CO2_ is stomatal conductance to CO_2_, and the factor 1.6 is the ratio of diffusivity of water vapor to CO_2_. δ^13^Catm and *ca* were obtained from (Mccarroll & Loader, 2004) for the period 1967-1997, and for the rest of the period values were obtained from Dr. Pieter Tans, NOAA/GML (gml.noaa.gov/ccgg/trends/) and Dr. Ralph Keeling, Scripps Institution of Oceanography (scrippsCO_2_.ucsd.edu/).

The oxygen isotope ratio (^18^O/^16^O) or δ^18^O was measured only for the northernmost site (MEL) and was also expressed in per mill with reference to the international Vienna Standard Mean Ocean Water (VSMOW). The precision of the instrument determined as the standard deviation of measurements using an internal wood standard was 0.25‰. Isotope corrections were done using linear regression of all δ^18^O data using two international standards (IAEA 601 and IAEA 602).

### 2.3 Data analyses

Temporal trends in Δ^13^C and iWUE were assessed using linear regressions and the Mann- Kendall non-parametric test in cases where data did not comply normality. Moreover, the three well-known theoretical scenarios for gas exchange under rising CO_2_ (constant ci, constant ci/ca and constant ca-ci) of Saurer et al. (2004) were used as a guideline to interpret the observed non-linear changes in iWUE over the study period. These scenarios differ only in the degree in which the increase in ci follows the increase in ca (either not at all, in a proportional way, or at the same rate, respectively). Mean ci over 1968–1979 was used as the starting point to evaluate the non-linear trends using generalized additive models (GAM). To determine shifts in the iWUE series, a Rodionov regime shift test (Rodionov, 2004) with a cut-off length of 10, a Huber’s weight parameter of 1 and a significance level of 0.05 was applied to series from each site using a MS Excel program (https://www.beringclimate.noaa.gov/regimes/).

In order to understand the relationship between tree growth and physiological responses as inferred from stable isotopes in tree rings, the Pearson’s correlation coefficient was used to relate the standardized tree-ring width chronologies reported by Urrutia-Jalabert et al., (2021) and Δ^13^ C and δ^18^O. In addition, regression analyses were used to relate the basal area increment (BAI), available from Urrutia-Jalabert et al., (2021), with iWUE. BAI chronologies help to reduce the age-size effect associated with the geometry of the stems, as well to preserve the low frequency variability (Biondi, 1999).

To determine the association between climate and the isotopic composition in tree rings, correlation coefficients were calculated between Δ^13^C and δ^18^O chronologies and precipitation and maximum temperatures. When the time series of tree rings or climate presented significant positive or negative trends though time, the residuals of the adjusted linear regression were used to obtain correlations without the influence of trends. The climate records used were regional averages (Maule, Bío Bío, Araucanía and Los Ríos regions) developed by Urrutia-Jalabert et al. (2021) from several stations. Records of annual or seasonal precipitation and maximum temperatures were calculated as normalized departures with zero mean and unit variance (z scores).

A bivariate response surface between tree-ring width variability and the joint variability of the carbon and oxygen isotopes (Barichivich et al., 2021) was used to aid the mechanistic interpretation of the coupled growth and physiological responses at the northernmost site under a drier Mediterranean climate (MEL). Following the procedure for the growth isotope tree-ring triplet of Barichivich et al. (2021), a smoothed response surface was fitted using a data-adaptive bivariate generalized additive model (GAM) implemented with the “mgcv” package (Wood, 2017) in the R environment (R Core Team, 2020).

## 3 Results

### 3.1 Carbon and oxygen isotopes chronologies

**Figure 2:**
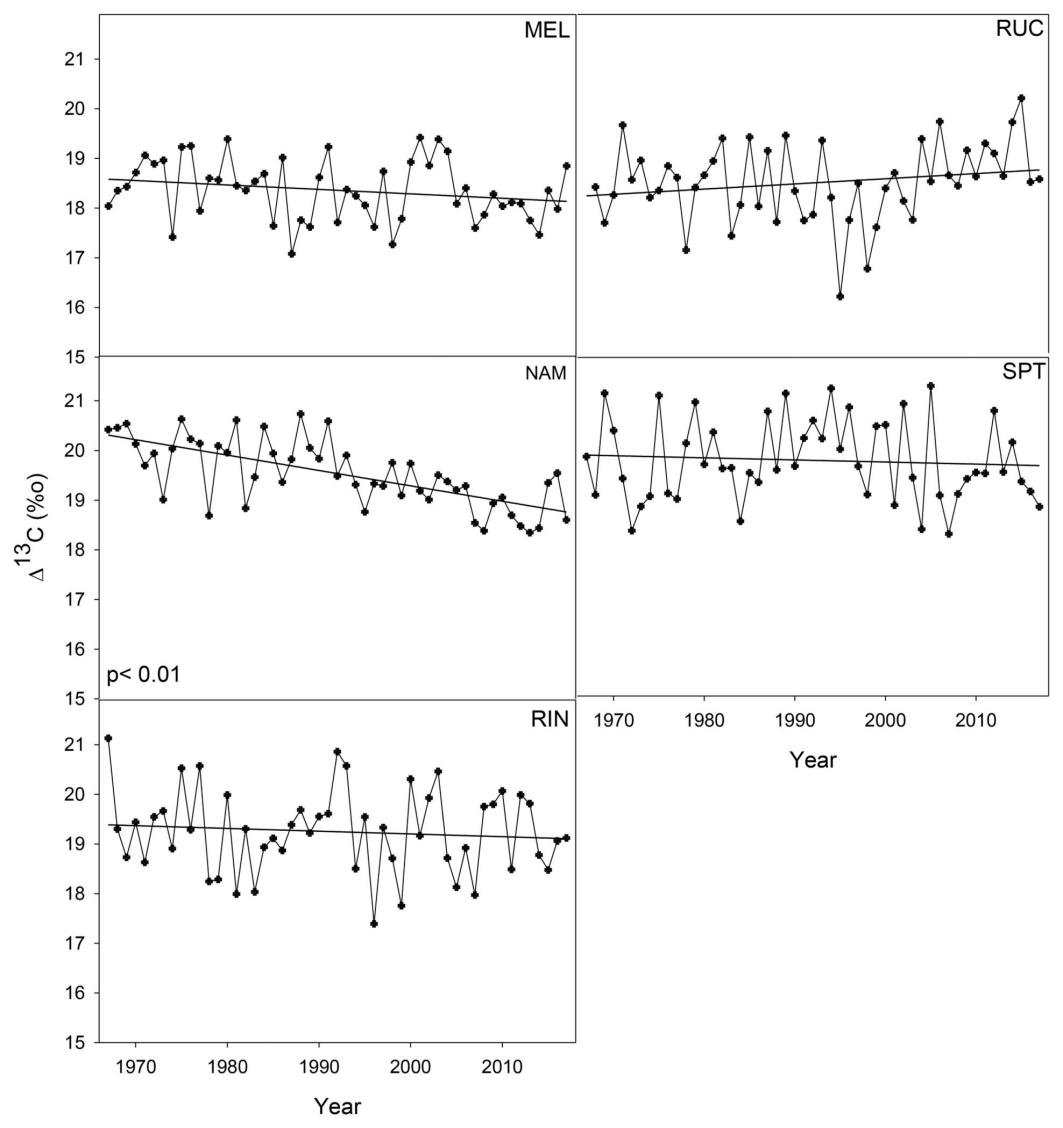
Annual carbon isotopes discrimination Δ^13^ C (‰) and fitted linear trends for the period 1967-2017 in five *Nothofagus obliqua* stands across a latitudinal gradient in south-central Chile. See Figure 1 for site abbreviations. The only significant negative slope for Δ^13^ C was found in the NAM site (R^2^adj: 0.46, *p*<0.01).

For most sites, Δ^13^C showed a negative trend, except for the second northernmost site RUC. However, only the mid-gradient site NAM showed a significant negative trend (*p*<0.01, Figure 2). Additionally, an apparent breakpoint in the Δ^13^C trend at NAM was detected in 1989 (Segmented Package, Muggeo, 2008).

iWUE increased significantly at all sites (Figure S1). The percent change in iWUE calculated as the mean of 2013-2017 compared to the mean of the early period 1968-1972, was not uniform across the gradient. It ranged from 14% in the second northernmost site RUC to 47% in the site south of it (NAM), with 25-28% increase in the two southernmost temperate sites (RIN and SPT, respectively) and 32% in the northernmost Mediterranean site (MEL).

**Figure 3:**
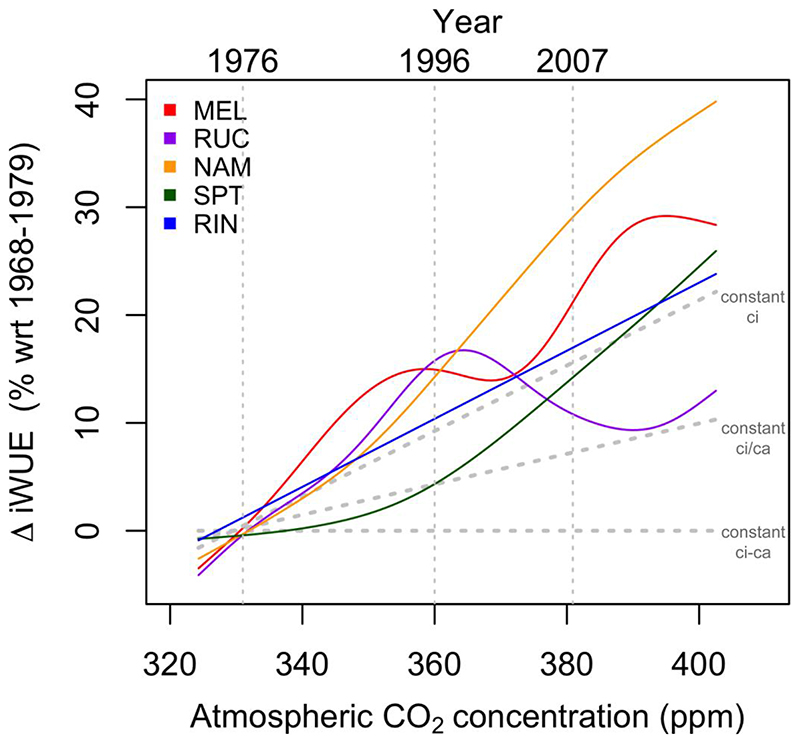
Change in iWUE with respect to the period 1968-1979 for the study sites. The curves represent the non-linear trends from a generalized additive model (GAM) fitted to the data. The change in iWUE under the three theoretical gas-exchange scenarios proposed by Saurer et al., (2004) are shown as dotted gray lines.

When non-linear trends in iWUE were extracted using the GAM adjustment and compared with the three response to CO_2_ scenarios proposed by Saurer et al., (2004), responses varied among sites. The mid-gradient NAM site outstood as the one with the steepest increase in iWUE since the 1990s with values well above the constant ci scenario (Figure 3). In addition, the iWUE of the northernmost site MEL was also above this scenario but showed a stabilization around the 1990s and an increase again around the 2000s (Figure 3). The second northernmost site RUC had a steep rise above the constant ci scenario until the mid-1990s and a decrease afterwards to finally reach the constant ci/ca scenario. Finally, the southern SPT site showed a slow increase between the constant ci-ca and ci/ca up to the mid-1990s and a steeper increase up to the constant ci scenario during recent years (Figure 3), and the southernmost RIN site showed a permanent increase similar to the constant ci scenario. When the Rodionov’s test was applied to find shifts in mean iWUE values, the MEL site showed significant shifts in 1987 and 2007, RUC in 1995, NAM in 1992 and 2007, SPT in 2004 and RIN in 1996 (Figure S1). Changes in mean values in the 1990s were observed in RUC, NAM and RIN, while MEL, NAM and SPT recorded the changes in mean values after the 2000s. No significant temporal trends in oxygen isotopes (δ^18^O) were observed in MEL for the period 1970-2017.

### 3.2 Tree growth and isotopes

**Figure 4:**
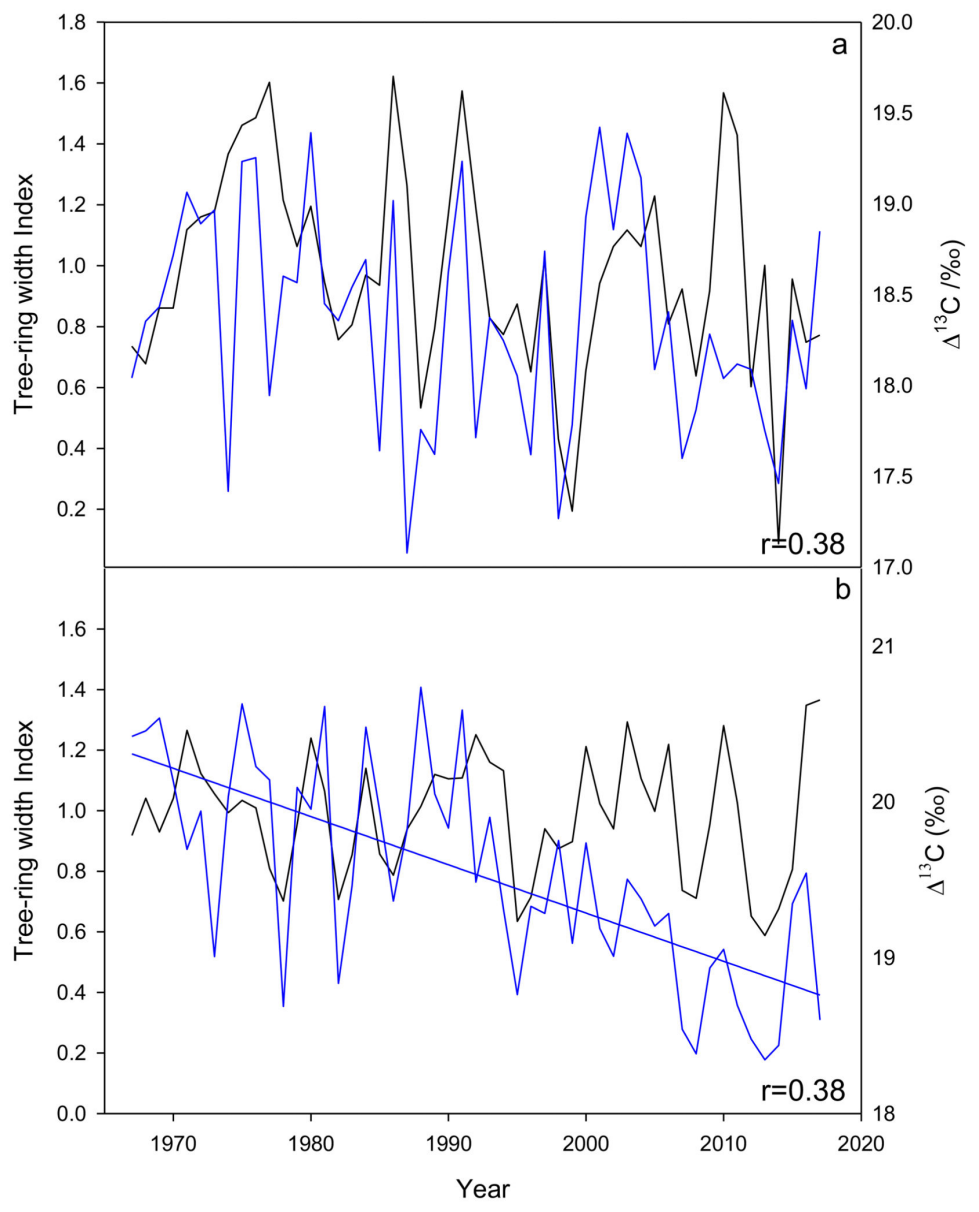
a) Significant correlations between the tree-ring width chronology (black) and Δ^13^ C (blue) at the MEL site (*p*<0.05); b) Significant relationship between the tree-ring width chronology (black) and Δ^13^ C (blue) at the NAM site (*p*<0.05).

**Figure 5:**
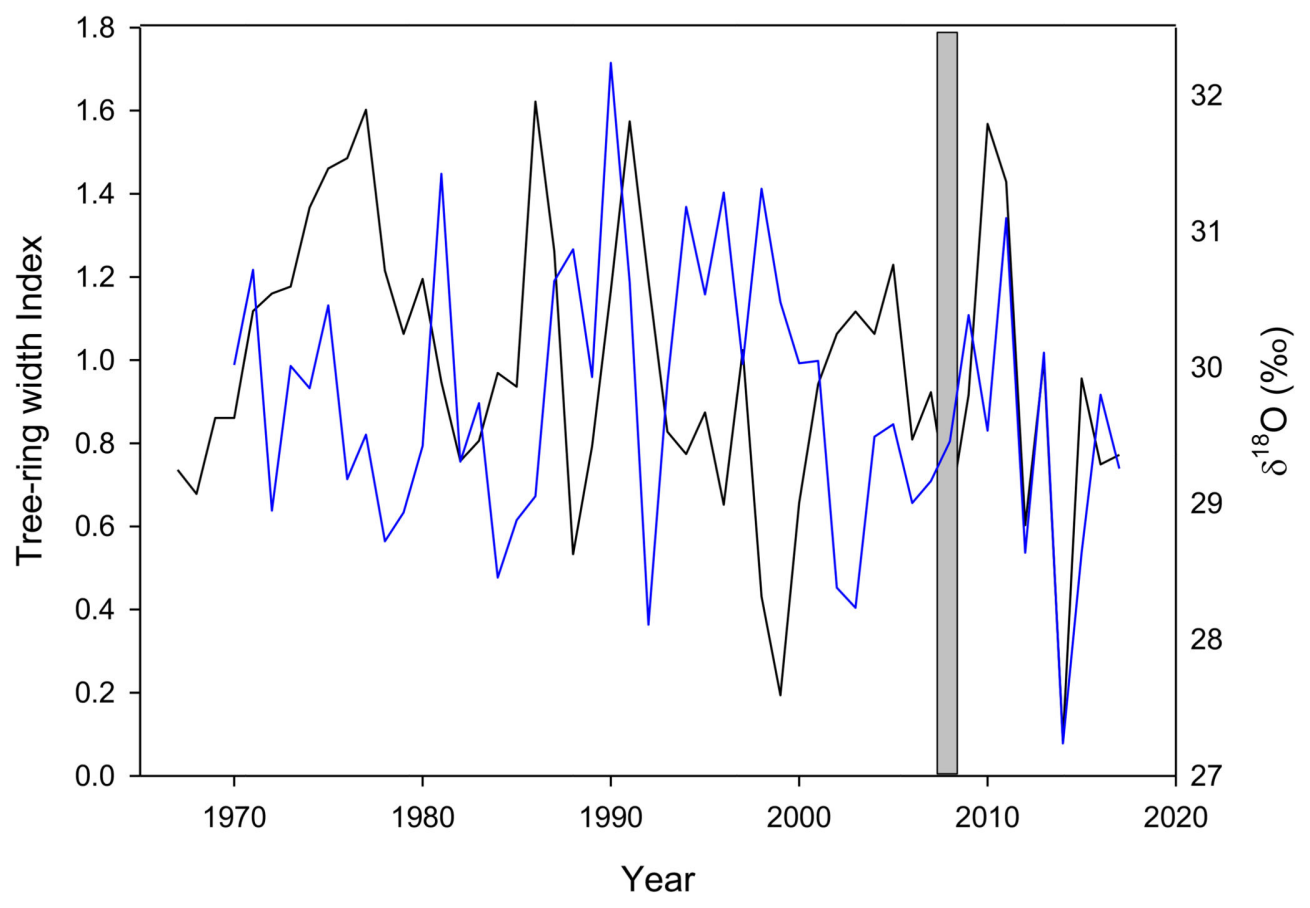
No significant relationship between tree-ring growth (black) and δ^18^O (blue) throughout the 1970-2017 period (r=0.02) at the MEL site. A positive and significant association was observed since 2007 (r=0.72, *p*<0.01), which had not previously been observed at this site.

Significant correlations between tree growth (standardized tree-ring chronologies) and Δ^13^C were only found in the northernmost site MEL and the mid-gradient site NAM (Figure 4). There was no significant relationship between tree growth and δ^18^O in MEL, but there was a clear change in their relationship since around 2007 (from r= -0.29, period 1970-2006, *p*>0.05 to r= 0.72, period 2007-2017, *p*<0.01). Since this date, low growth coincided with low enrichment δ^18^O (Figure 5).

**Figure 6:**
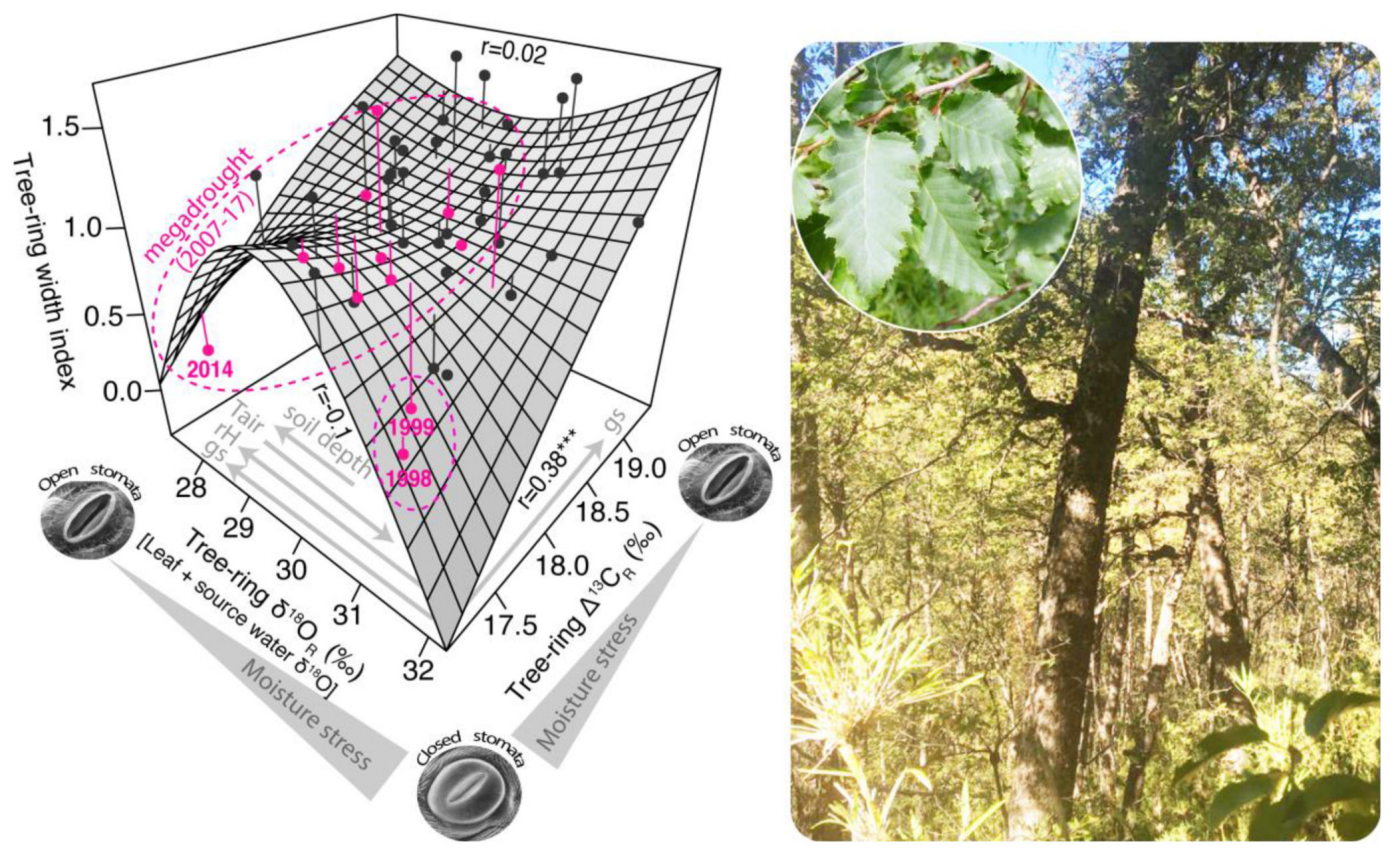
The growth-isotope tree-ring triplet (left) and view of the leaves and forest stand (right) of *N. obliqua* at the northernmost site (MEL). The three-way correlations between the tree-ring variables are indicated along the edges of the surface: tree-ring width vs δ^18^O (top), tree-ring width vs △^13^C (right) and δ^18^O vs △^13^C (left). The asterisks denote the significance levels of the correlations: * p<0.1, ** p<0.05 and *** p<0.001. The relationship between stomatal opening and moisture stress is indicated along the isotopic axes together with the expected changes in stomatal conductance (gs) and relative humidity (rH) according to the isotope model of Scheidegger et al. (2000). The expected enrichment/depletion of δ^18^O with increasing air temperature (Tair) and soil depth at the site is also indicated. The individual years are indicated by the dots and their vertical distance to the surface is shown by the line. The drought years are indicated in magenta. The position in the triplet of the extreme drought years 1998 and 1999 indicates that low growth was associated with stronger stomatal closure as expected under water limitation. In contrast, tree growth did not decrease and the isotopic signals indicate little stomatal limitation of gas exchange during the megadrought (2007-2017).

The bivariate surface response of tree-ring width as a function of the variability of both isotopes at the northernmost site MEL reveals the relationships of tree growth with environmental variability (temperature and drought stress) and stomatal responses, and also deviations from expectation in these water-limited locations that can be attributed to changes in source water (Figure 6). The resulting growth-isotope triplet shows that ring width has a positive and significant linear relationship with Δ^13^C (*r* = 0.38, *p* < 0.001) but no significant linear relation with δ^18^O, suggesting that growth is moderately coupled with stomatal activity. However, the overall isotopic variability is not intercorrelated (*r* = 0.10, *p* > 0.05) and the pattern of δ^18^O variability in tree rings suggests a changing influence from variations in source water δ^18^O during the megadrought since 2007. For instance, extreme drought years prior to the megadrought in 1998 and 1999 map into the tree-ring triplet as years of extremely low growth associated with the highest stomatal closure (i.e., highly enriched δ^18^O and low carbon discrimination) as it would be expected under dry conditions.

In contrast, during the megadrought (2007-2017) tree growth has not declined, and the isotopic variability indicates low stomatal limitation of gas exchange. Only in 2014 tree growth declined, but it was associated with very low stomatal closure. The contrasting growth-isotope response between the protracted megadrought and the extreme droughts in 1998-1999 indicates that trees have shifted their water use to deeper, less enriched source water in the soil profile in recent years, as an acclimation to the megadrought.

**Figure 7:**
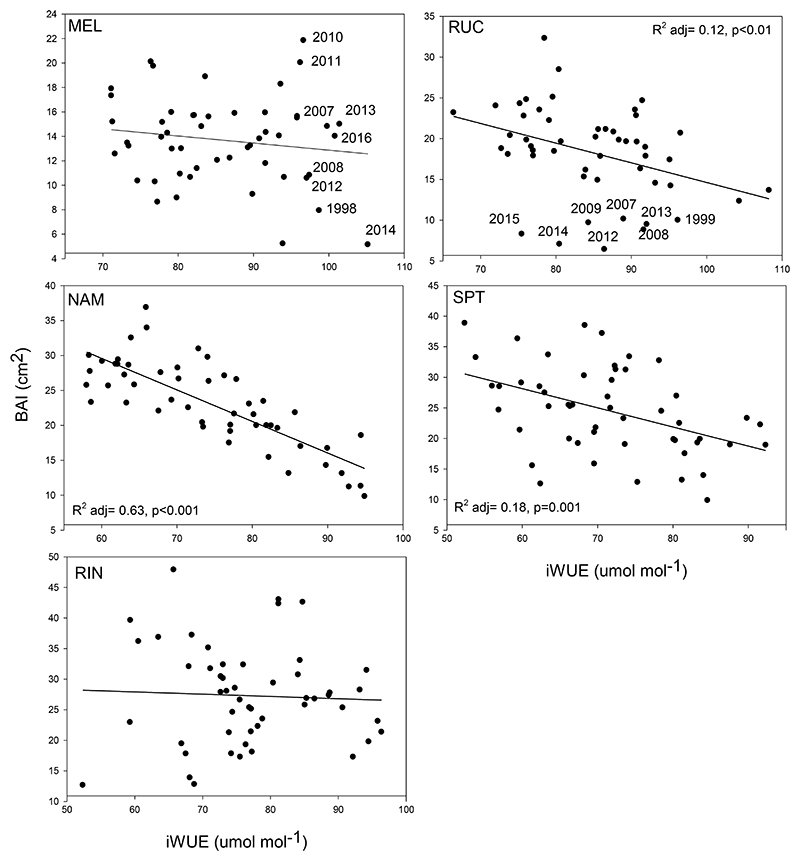
Relationship between mean yearly BAI (cm^2^) and intrinsic water use efficiency (iWUE) in trees from the different sites for the period 1967-2017. See Figure 1 for site abbreviations. Only significant relationships (adjusted R^2^ for the regressions and significant *p* values) are shown. Some individual years since 2007 are shown denoting high iWUE values, but not an overall reduction in tree growth in MEL, and low BAI values not associated with high iWUE in RUC.

Negative relationships between iWUE and BAI (cm^2^) were only significant in RUC, NAM and SPT (Figure 7). In the mid-gradient site NAM, increases in iWUE were strongly negatively associated with a decrease in BAI (Figure 7). For the northernmost site MEL and the southernmost site RIN, increases in iWUE during recent decades were not associated with reductions in tree growth. For MEL, the largest iWUE values in the whole studied period (>96 umol mol^-1^) were observed in recent years (since 2007) and they were associated with very high (years 2010 and 2011) and very low BAI values (year 2014, Figure 7). For the second northernmost RUC site, it is interesting to note that almost all the years with low BAI were since 2007 and that they were not associated with high iWUE values.

### 3.3 Climate-isotopes relationships

**Figure 8:**
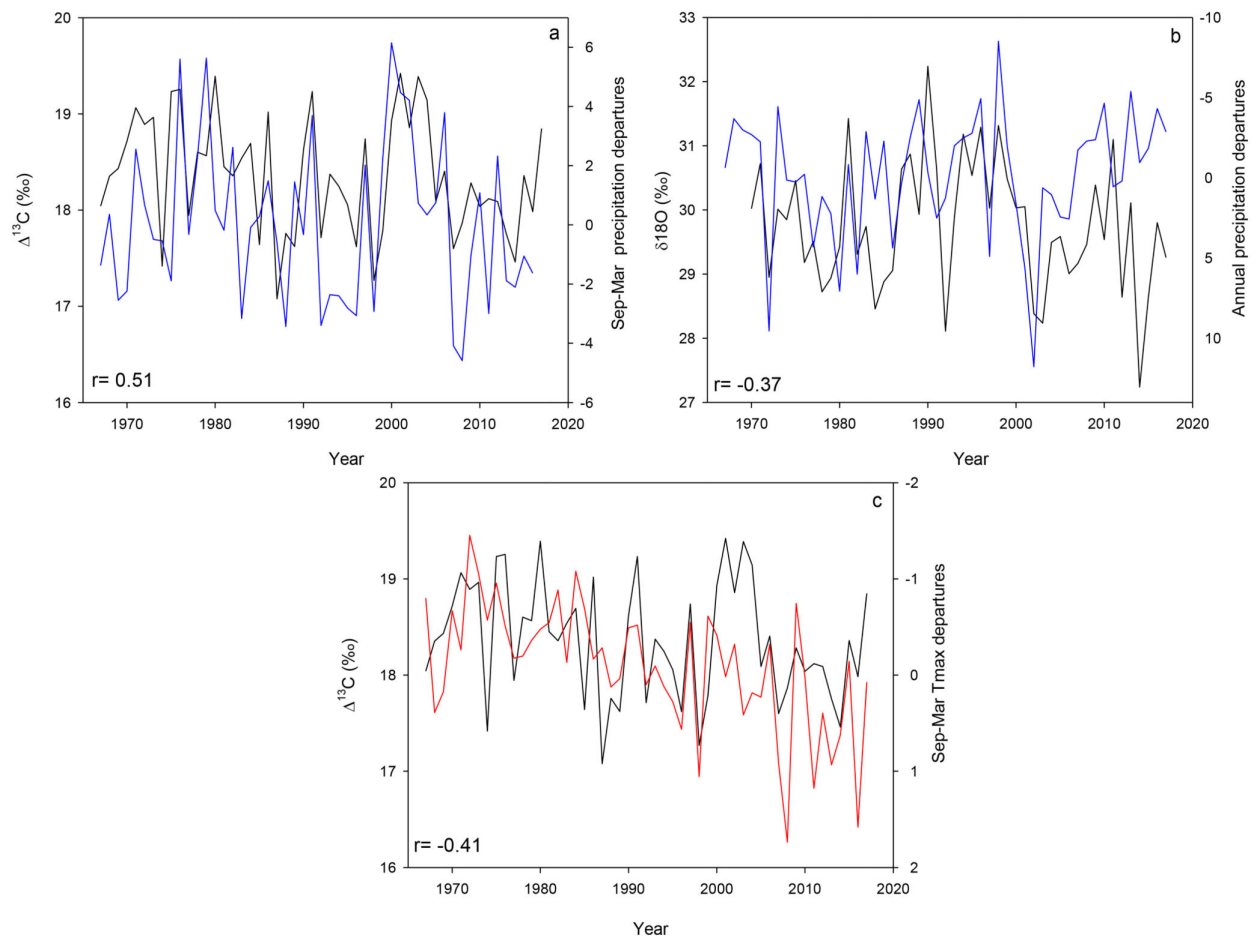
Significant correlations between isotopic signatures and climate at MEL: a) Relationship between Δ^13^ C (black) and growing season (September-March) precipitation (blue, *p*<0.05); b) relationship between δ^18^O (black) and annual precipitation (blue, axis inverted, *p*<0.05); and c) relationship between Δ^13^ C (black) and growing season maximum temperatures (September-March, red, *p*<0.05).

The most important significant relationships between isotopic signatures and climate variability were found at the northernmost site MEL. Δ^13^ C from MEL was positively related to spring-summer precipitation (*p*<0.01) and negatively related to spring-summer maximum temperatures (Figure 8 a,c). δ^18^O isotopic variability was negatively related to annual precipitation. However, since around mid 2000’s, precipitation decreased considerably and δ^18^O instead of increasing, it decreased (Figure 8b). In fact, the correlation coefficient increased from r= -0.37 to r= -0.52, when the period 2007-2017 was excluded.

The positive correlation coefficient between the standardized tree-ring width chronology from MEL and annual precipitation increased when only the 1967-2006 period was considered, compared to when the whole 1967-2017 period was included (from r= 0.36 to r= 0.44, p<0.01, Figure S2). This means that the relationship between tree-ring width and rainfall was weaker after 2006, indicating a change in the forest environmental response since 2007.

In the other stands, Δ^13^C was positively correlated with growing season precipitation (September-March) only in the southern site SPT (r=0.35, p<0.01). Significant correlations between iWUE and climate were found with maximum temperatures in the mid-gradient site NAM and the northernmost site MEL and with precipitation in SPT (Figure S3).

## 4 Discussion

### 4.1 Tree-ring isotopes and growth

Stable carbon isotopes in tree rings of second-growth *N. obliqua* forests along the Mediterranean-Temperate biome transition indicated increasing iWUE and thus water savings since 1967. Trees are discriminating less against ^13^C (Δ^13^C) over time, though one of the northern sites (RUC) showed the opposite trend. Additionally, trees in the midgradient at NAM showed the most pronounced negative trend in Δ^13^C showing an additional breakpoint to a steeper negative trend after 1989. This breakpoint could have been triggered by the dry conditions recorded in south-central Chile during that year (Urrutia-Jalabert et al., 2021) and is likely the driver of the latter change in iWUE mean conditions since 1992. The significant reduction in Δ^13^C at NAM, led to the strongest observed increase in iWUE (47%), value that is among the highest reported for different forests in the world throughout the entire twentieth century (Adams et al., 2020; Mathias & Thomas, 2021; Saurer et al., 2014), although higher values have been reported for shrubs (Kannenberg et al., 2021). The increase in iWUE at the northernmost site MEL is around the values reported for other tree species in Chile (Tognetti et al., 2014; Urrutia-Jalabert et al., 2015) and the low value reported for the second northernmost site RUC demonstrates that trees at this site are not adjusting to increases in atmospheric CO_2_ and/or drier conditions. Trees are even decreasing their iWUE since approximately 1995, showing less acclimation capacity to environmental changes. This behavior has also been reported for several species worldwide (Adams et al., 2020; Camarero & Fajardo, 2017; Saurer et al., 2014). RUC has the highest stand density among the studied forests, with 1100 trees ha^-1^ (Urrutia-Jalabert et al., 2021). A decrease in photosynthetic rates, associated with the increase in competition intensity and decrease in light availability in this very dense secondary stand, could have influenced this behavior (Martín-Benito et al., 2010; Park et al., 2018). This change could also have been triggered by dry conditions observed during 1995-1996 in south-central Chile, making the trees less efficient to assimilate carbon (Urrutia-Jalabert et al., 2021). In fact, the years with the lowest BAI, mostly occurring since 2007, were not associated with higher water use efficiency. Niccoli et al., (2020) showed that a high intensity thinning increased tree growth and iWUE in the temperate deciduous species *Quercus robur*, due to a reduction in competition intensity and an enhanced photosynthetic activity under non-water limited conditions. In contrast, increases in stomatal conductance far prevailed in water-limited environments over increases in assimilation rates when the inter-tree competition was reduced (Giuggiola et al., 2016). RUC was ranked among the sites with the lowest drought tolerance in Urrutia-Jalabert et al., (2021). Besides a possible negative effect of increased stand density (i.e. high inter-tree competition) on tree growth (BAI) and physiological performance, it is not clear why the forest at RUC could not be acclimating to drier and warmer conditions, so future prospections at local soil and microclimatic conditions could shed light on this.

It is important to note that iWUE trends of *N. obliqua* at the different sites were mostly close to the constant ci scenario proposed by Saurer (except for SPT and RUC, before and after the mid-1990s, respectively), showing an active gas-exchange response of trees to the increase in atmospheric CO_2_ concentrations. This is consistent with what was reported for a long-lived conifer in southern Chile (Urrutia-Jalabert et al., 2015), but in contrast to what was reported for *N. dombeyi* and *N. betuloides* in the same southern region of Chile (Camarero & Fajardo, 2017). It is also important to highlight that all sites had shifts in mean iWUE values in the 1990’s or 2000’s, which coincided or were around drought years recorded in the region (e.g. 1995, 1996, 2007, Urrutia-Jalabert et al., 2021). Accordingly, increases in iWUE associated with drought events have been reported before (Jansen et al., 2013; López et al., 2021).

The only two sites with a positive association between standardized tree growth and Δ^13^C were the northernmost site MEL and the mid-gradient site NAM, demonstrating that when trees grow less at these sites, they also discriminate less against ^13^C as expected. This pattern it typically found in water-limited mid-latitude forests (Barichivich et al., 2021; Shestakova et al., 2019), where trees tend to reduce their stomatal conductance under dry conditions (Hereş et al., 2014; Timofeeva et al., 2017; Voelker et al., 2014).

The sites where increases in iWUE have been significantly associated with reductions in BAI were not only RUC, but also the mid-gradient site NAM and the southern site SPT, implying that despite trees being more efficient in their use of water, they are growing less through time. This has been reported in diverse forests around the world and mostly explained by a reduction in stomatal conductance produced by a temporal shift towards more stressful climate and site conditions (Lévesque et al., 2014; Urrutia-Jalabert et al., 2015; Zalloni et al., 2019). In this study we cannot discard that stand development (increasing competition), may influence the observed decrease in BAI (Urrutia-Jalabert et al., 2021), although this pattern was not observed in the also young stand RIN.

Finally, trees at the northernmost site MEL are not decreasing their growth during recent decades (Urrutia-Jalabert et al., 2021) and were also reported to be among the most resilient *N. obliqua* stands to droughts occurred during the past decades (Urrutia-Jalabert et al., 2021). δ^18^O at this site was not strongly related to tree growth during the analyzed period. However, this lack of relationship changed to a positive and significant one during the last ten years (2007-2017, see next section).

### 4.2 Tree-ring isotopes indicate a shift to a deeper soil water usage in the Mediterranean forest stand during the megadrought

The strongest associations between climate and Δ^13^C were found in the northernmost, driest, and more Mediterranean forest at MEL. Δ^13^C was positively related to growing season precipitation and negatively related to growing season maximum temperatures, suggesting that warmer and drier conditions could have reduced stomatal opening, making trees discriminate less against ^13^C as expected in water-limited environments (Voelker et al., 2014; Barichivich et al., 2021).

At the mid-gradient site NAM, where BAI is decreasing, significant correlations were found between iWUE and maximum temperatures. In this site, trees are being much more efficient in their use of water, probably due to increased summer temperatures, which are causing trees to close more their stomata (Helle & Schleser, 2004). It is likely that local soil conditions are especially limiting at this rainy place, since strong increases in iWUE and decreases in BAI were not seen in SPT which has even a higher basal area, but is located in a deeper soil and close to a stream (Urrutia-Jalabert et al., 2021).

δ^18^O variability at MEL showed a substantial increase in its negative correlation with annual precipitation, when the period 2007-2017, the same period for which the correlation with tree growth increased, was not considered (change from r= -0.37 to -0.52). Such changes in correlations indicate that tree growth and δ^18^O have been coupled during the megadrought, but that rainfall does not influence this pattern. This was supported by a weaker relationship between the tree-ring width chronology and rainfall when considering the same 2007-2017 period (r=0.36, *p*<0.01) rather than the period prior to 2007 (1967-2006, r=0.44). It is important to mention that the correlation previously reported for the whole overlapping period between tree-ring width and climate (1930-2017) was also r= 0.44 (Urrutia-Jalabert et al., 2021). These responses suggest that trees probably changed their water source since around 2007, and that they are probably using water from deeper moisture pools, less enriched in ^18^O than surface soil water (Barbeta et al., 2015; Querejeta et al., 2021; Sargeant & Singer, 2016; Sarris et al., 2013). Intensified soil and atmospheric dry conditions during recent decades, including the megadrought period, may have progressively reduced shallow soil water sources at the northernmost site MEL, so trees are exploring deeper soil layers to satisfy their water demands and maintain growth rates. Under decreasing surface soil moisture availability, deeper water pools can be used by trees (David et al., 2007; Kurz-Besson et al., 2006), producing a detectable decline in tree-ring δ^18^O, thus masking the evaporative enrichment trends from leaf water (Sarris et al., 2013). Deeper soil water reserves may form from surplus winter precipitation integrated over several years, and may be available for trees at some point later in the season (Sarris et al., 2007; Schenk & Jackson, 2002). The tree-ring triplet also shows an opposite behavior, especially in δ^18^O, during severe droughts occurring before 2007 (1998-1999) and after 2007 (e.g., 2014). This usage of deeper water has allowed *N. obliqua* trees in the drier Mediterranean region to keep their radial growth rates constant during recent years, suggesting that landscape heterogeneity in soils exerts an important modulation of forest responses to increasing droughts.

However, extreme droughts occurring in Mediterranean climates may aggravate persistent dry conditions, like what has occurred during the megadrought, leading to an eventual depletion of deeper water reservoirs and the consequent negative effects on vegetation (Barbeta et al., 2015). In addition, transient shifts to usage of deeper water sources may cause a reduction in nutrient uptake (nutrients are present in the topsoil) and carbon assimilation, leading anyway to persistent reductions in growth and iWUE in the long term (Querejeta et al., 2021). A recent study in Argentina demonstrated that *Nothofagus dombeyi* trees that died due to droughts used deeper soil water sources and also showed a poorer nutritional status than surviving trees (González de Andrés et al., 2021).

Most of the previous tree-ring studies in the region, have shown decreasing growth trends in different species during recent decades or during the megadrought period (Fajardo et al., 2019; Venegas-González et al., 2022; Villalba et al., 2012). A very closely related species growing in central Chile (*Nothofagus macrocarpa*), is already showing negative growth trends especially since the 1980’s (Venegas-González et al., 2018, 2019). Our results demonstrate for the first time that forests in the region might be able to maintain radial growth rates and actively acclimate to persistent droughts by tapping deeper water sources. This finding implies that in the mid-term, forests through the region will likely show heterogenous responses to worsening drought, depending on the availability of landscape refuges with deeper soil water reservoirs and the functional capacity of the species to access them by investing in deep roots. Modelling studies should help disentangling these isotopic and physiological responses observed at the drier Mediterranean sites and project the fate of these forests in a drier future.

## 5 Conclusions

Despite sharing similar warming and drying trends across the latitudinal gradient in south-central Chile, not all *N. obliqua* stands are physiologically responding the same way. All stands increased their iWUE during the studied period (1967-2017), but values fluctuated between 14% at the second northernmost site RUC and 47% at the mid-gradient site NAM. In fact, trees from RUC are even decreasing their iWUE since the mid-90s, probably due to a high inter-tree competition and associated light limitation that reduces photosynthetic rates. Only two out of the five stands (MEL and NAM) presented significant correlations between isotopes and tree growth demonstrating an interannual coupling between woody growth and carbon assimilation. Furthermore, mainly MEL, and to a lesser extent NAM and the southern site SPT, showed significant correlations between Δ^13^C and/or iWUE and climate variables. These results indicate a non-uniform response of *N. obliqua* second-growth forests to climate change, being their response strongly modulated by local site and soil conditions. Moreover, it was noteworthy to find that the northernmost Mediterranean stand MEL, changed its δ^18^O trajectory during the megadrought period, and trees seem to be acclimating to drier conditions by tapping water from deeper soil layers. Further studies should assess what has been the response of this stand to the “Hyper drought” occurred in 2019, since the megadrought still continues until today. It is also important that future treering studies in the Mediterranean-temperate climate transition in Chile include combined carbon and oxygen isotopes, to fully disentangle the physiological processes behind the observed growth patterns. Finally, it is essential to complement tree growth studies with soil and ecophysiological sampling to better predict the diversity of forest responses to climate and environmental changes.

## Data Availability

Data used in this study will be ready available for reviewers upon request and will be shared in a public repository if the paper is published.
